# Localized Pulmonary Vein Scar Promotes Atrial Fibrillation in High Left Atrial Pressure

**DOI:** 10.3389/fphys.2021.709844

**Published:** 2021-08-25

**Authors:** Lisa A. Gottlieb, Fanny Vaillant, Emma Abell, Charly Belterman, Virginie Loyer, Dounia El Hamrani, Jérôme Naulin, Marion Constantin, Bruno Quesson, Bastiaan J. Boukens, Ruben Coronel, Lukas R. C. Dekker

**Affiliations:** ^1^IHU Liryc, Electrophysiology and Heart Modeling Institute, University of Bordeaux, Bordeaux, France; ^2^AUMC, Academic Medical Center, Department of Experimental Cardiology, Amsterdam, Netherlands; ^3^AUMC, Academic Medical Center, Department of Medical Biology, Amsterdam, Netherlands; ^4^Department of Electrical Engineering, University of Technology, Eindhoven, Netherlands; ^5^Department of Cardiology, Catharina Hospital, Eindhoven, Netherlands

**Keywords:** atrial fibrillation, pulmonary vein, myocardial scar, atrial stretch, left atrial pressure, tissue excitability

## Abstract

**Background:**

Pulmonary vein (PV) ablation is unsuccessful in atrial fibrillation (AF) patients with high left atrial (LA) pressure. Increased atrial stretch by increased pressure is proarrhythmic for AF, and myocardial scar alters wall deformation. We hypothesized that localized PV scar is proarrhythmic for AF in high LA pressure.

**Methods:**

Radiofrequency energy was delivered locally in the right PV of healthy sheep. The sheep recovered for 4 months. Explanted hearts (*n* = 9 PV scar, *n* = 9 controls) were perfused with 1:4 blood:Tyrode’s solution in a four-chamber working heart setup. Programmed PV stimulation was performed during low (∼12 mmHg) and high (∼25 mmHg) LA pressure. An AF inducibility index was calculated based on the number of induction attempts and the number of attempts causing AF (run of ≥ 20 premature atrial complexes).

**Results:**

In high LA pressure, the presence of PV scar increased the AF inducibility index compared with control hearts (0.83 ± 0.20 vs. 0.38 ± 0.40 arb. unit, respectively, *p* = 0.014). The diastolic stimulation threshold in high LA pressure was higher (108 ± 23 vs. 77 ± 16 mA, respectively, *p* = 0.006), and its heterogeneity was increased in hearts with PV scar compared with controls. In high LA pressure, the refractory period was shorter in PV scar than in control hearts (178 ± 39 vs. 235 ± 48 ms, *p* = 0.011).

**Conclusion:**

Localized PV scar only in combination with increased LA pressure facilitated the inducibility of AF. This was associated with changes in tissue excitability remote from the PV scar. Localized PV ablation is potentially proarrhythmic in patients with increased LA pressure.

## Introduction

Atrial fibrillation (AF) is a common cardiac arrhythmia that afflicts approximately 10% of the population above 80 years (Kannel and Benjamin, 2009). The underlying mechanisms of AF are multifold and complex, including stretch of the atrial myocardium (Schotten et al., 2011). In fact, Heart failure, hypertension, and valvular disease lead to increases in atrial pressure and are associated with AF (Kannel and Benjamin, 2009). Increased AF inducibility and heterogeneous shortening in atrial refractoriness and prolongation of conduction are observed in the experimental setting of atrial stretch (Ravelli and Allessie, 1997; Eijsbouts et al., 2003; Ravelli et al., 2011). Also, stretch of the pulmonary veins (PVs) increases the rate of spontaneous activation (Chang et al., 2007).

Ectopy from the PVs is considered to trigger paroxysmal AF (duration of AF episodes < 1 week) (Haissaguerre et al., 1998). Therefore, electrical isolation of the PVs from the left atrium (LA) by ablation is recommended in paroxysmal AF patients who do not respond to pharmacological therapy (Calkins et al., 2018). However, in 40% of these patients, a single PV isolation ablation procedure does not prevent AF on the long term (Kis et al., 2017). Particularly, a preexisting large LA, comorbidity of hypertension, and a LA pressure > 15 mmHg measured during the ablation procedure are risk factors for ablation failure (Themistoclakis et al., 2008; Evranos et al., 2016; Sramko et al., 2017). This questions the role of high atrial pressure on ablation success.

During the PV ablation procedure, ablative energy is applied locally to the PV myocardium (Calkins et al., 2018). The ablated sites develop into scar tissue in the course of weeks to months (Gottlieb et al., 2021). Myocardial scar alters the wall movement and strain of the myocardium in proximity to the scar (Ashikaga et al., 2005; Nori et al., 2009; Bertini et al., 2010). Such regional mechanical changes increase the heterogeneity in wall deformation and are proven to be proarrhythmic in the setting of ventricular infarction scar (Bertini et al., 2010).

We hypothesized that the wall motion changes associated with PV ablation scar can be proarrhythmic, particularly when stretch is increased by high atrial pressure. We created a localized scar in the right PV (RPV) of healthy sheep and studied the inducibility of AF in an *ex vivo* working heart setup during low and high LA pressure. We demonstrated that a PV scar is proarrhythmic in high LA pressure.

## Materials and Methods

The study was carried out in accordance with the EU Directive 2010/63/EU for the protection of animals used for scientific purposes and approved by the local ethical authorities at the University of Bordeaux, France (approval number 7995). Conventional safety measures were ensured during the entire experimentation.

### Catheterization Procedure

**FIGURE 1:**
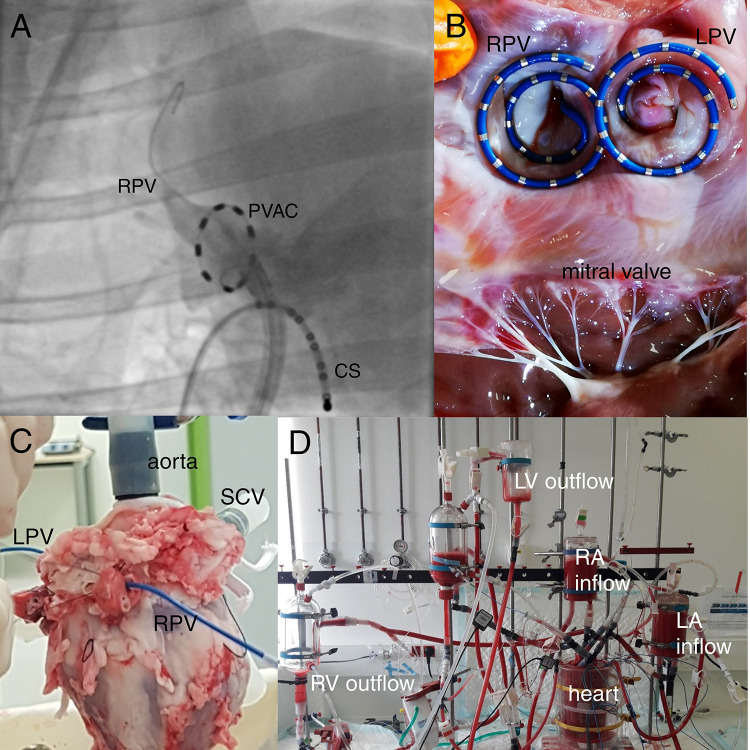
*In vivo* and *ex vivo* methodology. **(A)** Fluoroscopic image (right anterior oblique view of 41°) with circular ablation catheter (“PVAC”) positioned in the right pulmonary vein (RPV) of a sheep. A catheter is placed in the coronary sinus (CS) for anatomical reference. **(B)** Diagnostic double-helix catheters positioned in the RPV and right pulmonary vein (LPV) of the explanted heart. The photo is taken after *ex vivo* experimentation. **(C)** The PVs were tied off with the diagnostic catheters in place. **(D)** The heart was connected to a four-chamber working heart setup and perfused with an autologous blood:Tyrode’s solution. A bucket with perfusion solution was placed around the heart. The atria were each connected to a reservoir “preload” chamber, and LA pressure was modulated by changing the height of the left atrium (LA) reservoir chamber. LV: Left ventricle. RA, right atrium; RV, right ventricle; SCV, superior caval vein.

Healthy female sheep (*n* = 13, 53 ± 5 kg, 2–3 years old, sheep strain: Charmoise) were catheterized through the femoral veins under sterile conditions and general anesthesia (i.e., premedication: 20 mg/kg ketamine + 0.1 mg/kg acepromazine, induction: 1 mg/kg propofol, maintenance: 2% isoflurane). LA access was achieved by transseptal puncture using a steerable sheath, and a circular multielectrode ablation catheter (PVAC Gold, Medtronic, Minneapolis, MN, United States) was placed in the RPV ostium under fluoroscopy guidance (Figure 1A). Radiofrequency energy was administered 2 × 60 s with 2:1 bipolar:unipolar phasing and a temperature limitation of 55°C. Two out of nine ablation electrodes were turned off to secure localized PV scar. The ablation reference electrode was placed on the lower back of the sheep. The animals recovered under surveillance in the animal facility for 1 week before returning to a hosting farm. One sheep died before follow-up, and 12 sheep were achieved for further analysis.

### Cardiac Magnetic Resonance Acquisition

Cardiac magnetic resonance (CMR) acquisition was conducted on a 1.5 Tesla system (MAGNETOM Aera, Siemens, Erlangen, Germany) with a 32-channel body coil and an 18-channel cardiac coil before and 4 months after the catheterization procedure (*n* = 12). The sheep were placed on their back on the scanner table under general anesthesia. Cine imaging was performed by ECG-gated steady-state free precession pulse sequence during forced breath-hold to acquire a transaxial stack with a slice thickness of 4 mm (*n* = 7) or 6 mm (*n* = 5). The slice thickness did not differ between the two acquisitions in the same animal.

The parameters used were as follows: field of view = 300 mm × 230 mm; matrix = 240 × 180; resolution in plane = 1.3 mm × 1.3 mm; flip angle = 58°; bandwidth = 992 Hz/pixel; echo time = 1.34 ms; repetition time = 21.98 ms; generalized autocalibrating partial parallel acquisition; acceleration factor of 3 with 75% partial Fourier acquisition.

The phase-contrast velocity-encoded images were acquired in a two-dimensional through-plane model to measure the mitral valve flow (Cochet et al., 2014). The post-ablation CMR was performed 120 ± 11 days after the pre-ablation CMR. The sheep recovered for a minimum of 4 days either before the ablative catheterization procedure or before the *ex vivo* experimentation.

### *Ex vivo* Heart Preparation

The sheep were sternotomized under general anesthesia (i.e., premedication: 20 mg/kg ketamine + 0.1 mg/kg acepromazine, induction: 1 mg/kg propofol, maintenance: 40 mg/kg/h ketamine + 2 mg/kg/h midazolam). Blood was collected through a peripheral vein during continuous saline infusion and halted if arterial blood pressure dropped below 50 mmHg. Ventricular fibrillation was induced by the retrograde infusion of a high potassium concentration cardioplegic solution (Custodiol HTK^®^, Cardiolink Group, Barcelona, Spain) in the aorta after clamping the heart vessels (Vaillant et al., 2016). The heart with intact pericardium and lungs was explanted and placed in 4°C saline solution.

The aorta, pulmonary artery, and superior caval vein were cannulated. The inferior caval vein was tied off. The RPV and left PVs (LPVs) were dissected. The sheep had 1 RPV, and 1 smaller superior and 1 larger inferior LPV. A 20-electrode spiral endocardial catheter (Inquiry AFocusII, St. Jude Medical, Saint Paul, MN, United States) was introduced into each of the RPV and the common antrum of the LPVs from the “pulmonary” side (Figure 1B). The PVs were tied off ensuring a fixed position of the catheters (Figure 1C). An incision was made in the LA appendage (LAA) in which a cannula was placed for supplying perfusion solution to the left heart. The heart was continuously perfused with the cardioplegic solution during the preparation time that lasted for 45–60 min.

The heart was connected to a perfusion system and retrogradely perfused through the aorta (Langendorff mode) with a whole autologous 1:4 blood:modified Tyrode’s solution (NaCl 118.0 mM, mannitol 16.0 mM, glucose 11.0 mM, NaHCO_3_ 25.0 mM, KCl 4.5 mM, MgCl_2_ 1.2 mM, NaH_2_PO_4_ 1.2 mM, CaCl_2_ 1.8 mM, sodium pyruvate 0.5 mM, lactate 1.0 mM), which was continuously oxygenized (95% O_2_, 5% CO_2_) and kept at 37°C. The cardioplegic solution was washed out, and the heart was defibrillated with the lowest possible energy (5–30 J). Spontaneous cardioversion to sinus rhythm occurred within the first 5 min of Langendorff perfusion in 2 PV scar hearts and 2 control hearts in which defibrillation was not needed. A bucket with perfusion solution and a reference electrode was placed around the heart. A pressure catheter was placed in the LA *via* the LAA cannula. Isosorbide dinitrate (4 mg; Risordan^®^, Sanofi Aventis, Gentilly, France) was given in the perfusion solution to prevent coronary artery spasms.

After 10 min of sinus rhythm, the perfusion system was switched from Langendorff to four-chamber working mode: The atria were each connected to a preload reservoir, and the ventricles connected each to an afterload reservoir (Figure 1D). Chamber pressures were monitored by a pressure catheter connected to a fluid-filled piezoelectric pressure transducer (IOX2 data acquisition system, EMKA Technologies, Falls Church, VA, United States). The LA pressure catheter was inserted into the LA *via* the LAA cannula. LA pressure was modulated by changing the height of the preload reservoir.

The PV stimulation protocols were carried out at a baseline LA pressure of 12 mmHg similar to that of conscious healthy sheep (Johnston et al., 1995), and known to be physiological in humans (Evranos et al., 2016; Sramko et al., 2017), as well as at a high LA pressure of 25 mmHg observed in patients with mitral valve stenosis and pulmonary hypertension (Walston and Kendall, 1973; Venkateshvaran et al., 2016). Twelve healthy female control sheep (50 ± 7 kg) without PV scar underwent similar *ex vivo* experimentation.

One heart with a PV scar did not recover from the explantation. We encountered after experimentation that, in 2 PV scar and 3 control hearts, the electrophysiological stimulation had been carried out in a baseline LA pressure lower than the study design permitted (< 10 mmHg), and these hearts were omitted from the analysis to ensure a comparable experimentation handling.

### Electrophysiological Experiment

**FIGURE 2:**
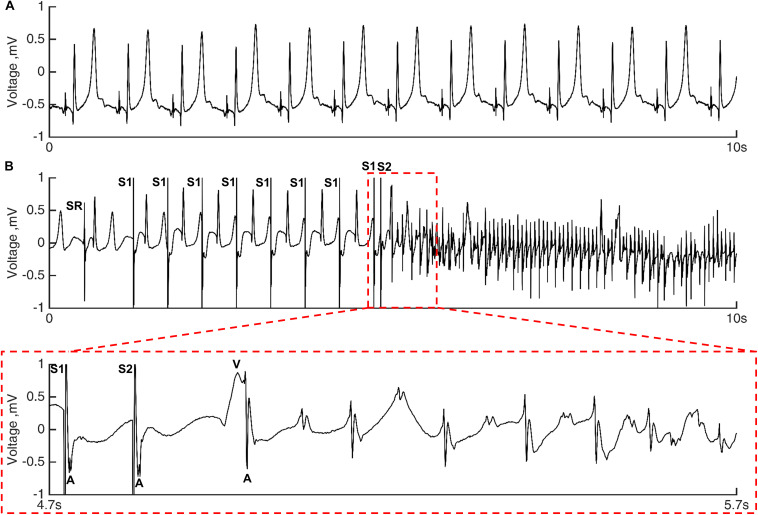
Unipolar electrograms tracings. **(A)** RPV electrograms during sinus rhythm in a heart with PV scar in high LA pressure. The hearts were in sinus rhythm before each induction attempt. **(B)** Tracing of atrial fibrillation (AF) induction in the same PV scar heart by premature stimulation in the LPV in high LA pressure. Local atrial and far-field ventricular activation are indicated with A and V, respectively. S1 denotes eight stimuli with a cycle length of 500 ms, and S2 marks the premature stimulus (S1–S2 coupling interval = 100 ms). The sinus rhythm (SR) occurring during the 1,000 ms pause before the first S1 stimulus is noted.

Unipolar electrograms were recorded and stored in Labsystem Pro (BARD EP, Boston Scientific, Marlborough, MA, United States; 1 kHz sampling frequency; filtering: low cutoff 0.05 Hz, high cutoff 500 Hz, adaptive notch filter; Figure 2A). A bipolar pacing protocol was carried out at 4 PV sites (2 in RPV and 2 in LPV) in both low and high LA pressure. First, the diastolic stimulation threshold was measured with 10 mA decremental steps, and the pacing was continued with 2 × threshold current. Then, the programmed S1–S2 stimulation with a single premature stimulus (8 × S1 of 500 ms cycle length followed by 1 × S2 with decreasing coupling interval beginning at 350 ms; 10 ms steps until the first loss of activation after which a 5-ms step continued by 1-ms steps; a 1,000-ms pause was inserted after each S2) was performed until the loss of local activation or AF (Figure 2B).

The refractory period was defined as the shortest S1–S2 coupling interval causing local activation and was only reached in programmed stimulation protocols without AF. The AF was defined as ≥ 20 premature atrial complexes (Larsen et al., 2015). In the case of AF, the atria were cardioverted with the lowest possible energy if necessary, and the heart was left to recover for 5 min in low baseline LA pressure before the next induction attempt at another site. A programmed S1–S2 protocol including thresholding qualified as an induction attempt of AF that therefore consisted of multiple series of S1–S2 stimuli until either AF or refractory period was reached. Maximum eight induction attempts were performed in each heart (2 in RPV and 2 in LPV in both low and high LA pressure). The heart was in sinus rhythm before each induction attempt.

### Histology

Three hearts subjected to radiofrequency delivery were preserved for histological evaluation after *ex vivo* experimentation. The RPV and LPVs were cut in the longitudinal direction; thus, each specimen included the atrial-PV junction and the distal PV. The specimens were fixated in paraformaldehyde (4%) at 5°C for a minimum of 2 weeks and dehydrated automatically (Leica HistoCore Pearl processor, Wetzlar, Germany) before being embedded in paraffin. Sections (6 μm thickness) were cut with a microtome and stained with Masson’s trichrome to visualize cardiomyocytes (red), nuclei (black), and collagen (green) before being digitally scanned with an objective 20 × and viewed in the software ZEN lite (Zeiss, Oberkochen, Germany).

### Cardiac Magnetic Resonance Analysis

**FIGURE 3:**
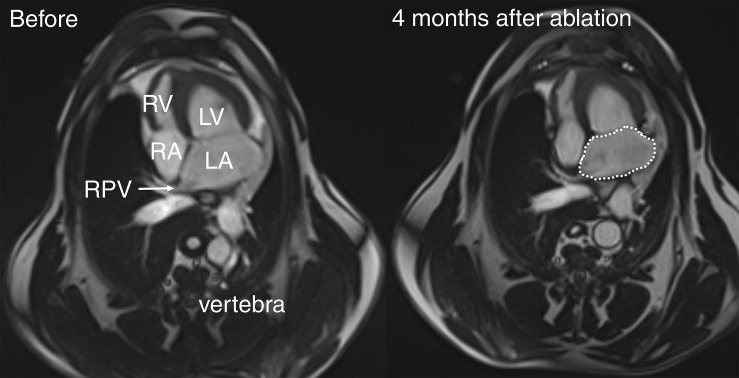
Cardiac magnetic resonance (CMR) images of sheep. Transaxial CMR images of sheep heart with RPV before (left panel) and 4 months after (right panel) ablative energy delivery localized in the RPV. The dotted line denotes the LA surface area for volume estimation. LV, left ventricle; RA, right atrium; RV, right ventricle.

The CMR images were only included in the study when pre- and post-ablation images were comparable, defined as including the same anatomical landmarks in similar stack slices and being without artifacts in the LA and PV regions (Figure 3).

The LA volume was measured at the moment of maximum LA dilatation and immediately before (pre-contraction) and after atrial contraction (minimum LA volume). The endocardial LA contours on each slice of the transaxial stack, while excluding the PVs and LAA, were tracked to obtain the LA area in the software Syngo.via [Siemens, Erlangen, Germany; Figure 3 (right panel)]. The volume was calculated as the sum of each LA area multiplied by the slice thickness.

We defined the LA active emptying fraction as (pre-contraction LA volume − minimal LA volume)/pre-contraction LA volume × 100% and the LA passive conduit fraction as (maximal LA volume − pre-contraction LA volume)/maximal LA volume × 100%.

### Electrophysiological Analysis

**FIGURE 4:**
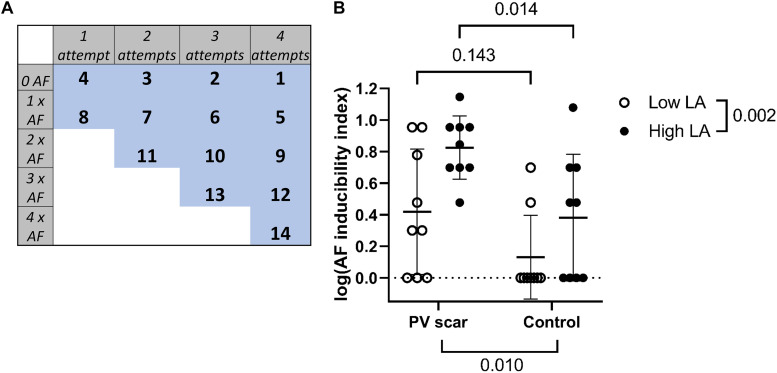
AF induction. **(A)** An AF inducibility index was calculated in low and high LA pressure in each heart based on the number of induction attempts performed and the number of attempts inducing AF. Maximum 4 induction attempts were performed in each pressure in each heart (2 in RPV and 2 in LPV). **(B)** The AF inducibility index in high LA pressure was significantly increased in PV scar hearts compared with control hearts (*n* = 9 vs. *n* = 9, respectively, *p* = 0.014; Bonferroni correction applied after a two-way repeated measures ANOVA. Overall PV scar effect *p* = 0.010 and overall LA pressure effect *p* = 0.002. A logarithmic transformation of the index values was performed before statistical testing.

The explanted hearts were not subjected to the same number of induction attempts due to a loss of ventricular contraction before the end of the experimental protocol (precluding the working heart mode). We, therefore, calculated an AF inducibility index in low and high LA pressure in each heart based on the number of induction attempts and the number of attempts causing AF (Figure 4A).

Similarly, means of diastolic stimulation threshold, S1–S2 coupling interval causing AF, and refractory period were calculated in each heart in low and high LA pressure. The heterogeneity of PV excitability was defined as the range in diastolic stimulation threshold including measurements from both PVs.

The unipolar electrograms were analyzed in a custom-made script in MATLAB (Mathworks, Natick, MA, United States). The maximum negative deflection of the unipolar electrograms was defined as the local activation. We measured the relative local activation times in the PVs during sinus rhythm and considered the temporal difference between the first and last activation as a measure of PV activation time.

### Statistical Analysis

The CMR parameters before and after ablation were compared with a two-tailed paired Student’s *t*-test or a Wilcoxon signed-rank test dependent on normality as tested with a Shapiro–Wilk test. The CMR data are expressed as mean ± SD or median (interquartile range) as appropriate.

A non-parametric Mann–Whitney *U* test was performed to test for a difference in S1–S2 coupling interval causing AF in hearts with and without PV scar [data expressed as median (interquartile range)]. The remaining nominal electrophysiological parameters were tested with a two-way repeated measures ANOVA using GraphPad Prism (GraphPad Software, San Diego, CA, United States). In case of an unbalanced design due to missing values, a mixed-effects linear model was performed. The Bonferroni correction was applied for multiple testing. These data are expressed as mean ± SD. A logarithmic transformation was applied to the AF inducibility index before statistical testing.

The site of stimulation (RPV vs. LPV vs. both RPV and LPV) causing AF in each heart was tested with a χ^2^ test of independence. Statistically significant differences were considered with *p*-values < 0.05.

## Results

### Localized Scar in the RPVs

**FIGURE 5:**
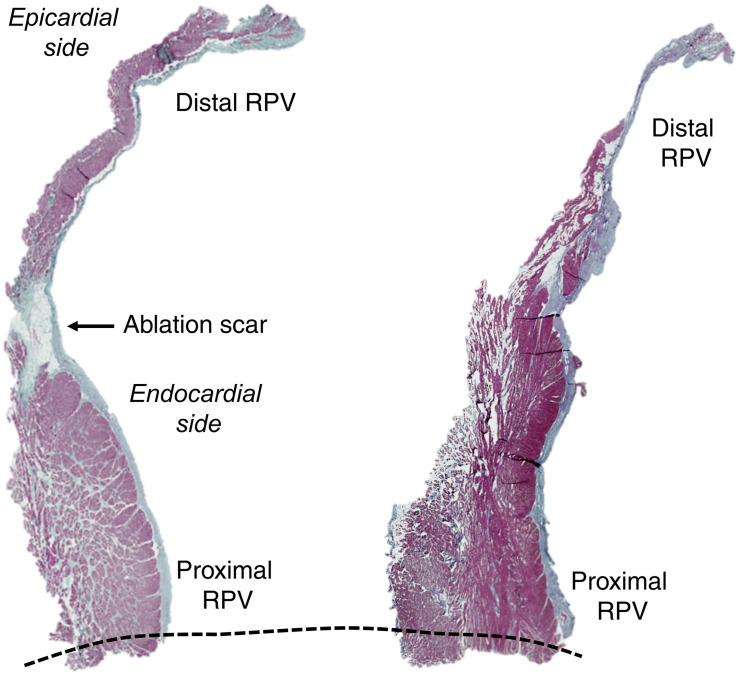
PV histology. Histological preparations of the RPV including the distal PV and the atrial-PV junction (dotted line) with Masson’s trichrome staining (red: cardiomyocytes, black: nuclei, green: collagen). The transmural collagenous scar tissue (arrow) in the left panel is noted. The right panel shows an RPV slice without a scar. The ablation catheter was advanced into the PV under fluoroscopic monitoring (see the position in Figure 1A).

Figure 5 (left panel) shows the histological images of the RPVs with acellular collagen accumulation. Not all RPV slices contained collagenous scar (Figure 5, right panel), which therefore occurred at localized areas within the RPV. The scar was not observed in the slices from the LPVs.

The LA volumes, the global active and passive LA functions, and the mitral valve flow were unaltered by the localized scar in the RPV (Table 1). We, therefore, concluded that the catheterization procedure with radiofrequency delivery only caused chronic changes locallly in the RPV.

**TABLE 1:** Left atrial (LA) volumes and functions before and after the creation of localized pulmonary vein (PV) scar.

	**Before**	**4 months after ablation**	**Before vs. after**
Maximum LA volume, ml	42 ± 7	42 ± 9	*p* = 0.752
Pre-contraction LA volume, ml	32 ± 7	32 ± 8	*p* = 0.628
Minimum LA volume, ml	27 ± 5	27 ± 7	*p* = 0.907
Active LA emptying fraction,%	14(7)	9(17)	*p* = 1
Passive LA conduit fraction,%	24 ± 10	24 ± 8	*p* = 0.932
Mitral flow, cm/s	64 ± 14	66 ± 12	*p* = 0.550
Sinus rate during CMR, bpm	72 ± 13	73 ± 15	*p* = 0.583

*Localized scar in the right pulmonary vein (RPV) neither alters the LA volumes nor alters the global functions (*n* = 11). One sheep was excluded from the analysis due to artifacts in the cardiac magnetic resonance (CMR) images. The data are expressed as mean ± SD or median (interquartile range) dependent on normality (tested with a Shapiro–Wilk test). Normally distributed data were tested with a paired Student’s *t*-test and non-normal data with a Wilcoxon signed-rank test.*

Then, we evaluated the effect of the localized PV scar on arrhythmogenesis in an *ex vivo* working heart setup under the condition of low baseline and increased LA pressure.

### Atrial Fibrillation Inducibility in Hearts With PV Scar

Figure 2B shows a tracing of AF induction with premature PV stimulation during high LA pressure in a heart with a PV scar. Due to a difference in induction attempts between the hearts, we calculated an AF inducibility index in each heart based on the number of induction attempts performed and the number of attempts inducing AF (Figure 4A). In high LA pressure, the presence of a PV scar increased the AF inducibility index compared with control hearts (0.83 ± 0.20 vs. 0.38 ± 0.40 arb. unit, *p* = 0.014; Figure 4B).

### Reduced Tissue Excitability in PVs With Scar

**FIGURE 6:**
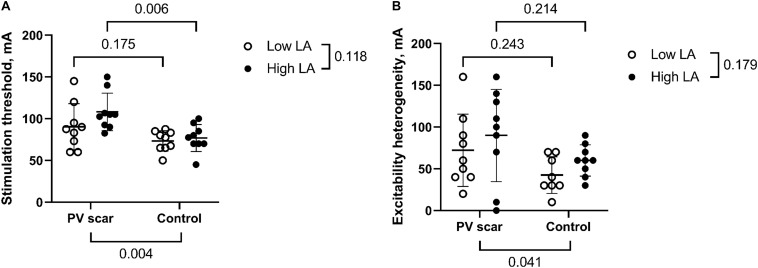
Diastolic stimulation threshold. **(A)** In high LA pressure, the diastolic PV stimulation threshold was higher in hearts with PV scar compared with control hearts (*n* = 9 vs. *n* = 9, respectively, a two-way repeated measures ANOVA). **(B)** The heterogeneity in PV excitability, defined as the range in stimulation threshold in both PVs, was larger in hearts with PV scar than in controls (*n* = 9 vs. *n* = 8, respectively, mixed-effects linear model). Of note, 1 control heart had only 1 induction attempt, and therefore 1 threshold measurement, in high LA pressure preventing calculation of a range in excitability.

The diastolic stimulation threshold in high LA pressure was increased in PV scar hearts compared with control hearts (108 ± 23 vs. 77 ± 16 mA, respectively, *p* = 0.006; Figure 6A). There was no difference in threshold between the PVs (*p* = 0.659), which indicates a remote electrical remodeling in the LPV in hearts with RPV scar. The heterogeneity in PV excitability was increased in hearts with PV scar compared with control hearts (low LA pressure: 72 ± 43 vs. 43 ± 22 mA; high LA pressure: 90 ± 55 vs. 60 ± 19 mA, respectively, PV scar effect *p* = 0.041, LA pressure effect *p* = 0.179; Figure 6B).

### Local PV Refractoriness

**FIGURE 7:**
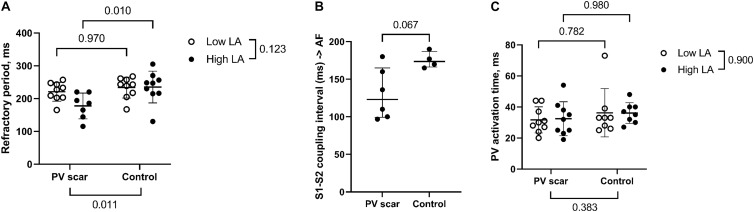
PV refractoriness and activation time. **(A)** The refractory period was reached in programmed stimulation protocols without AF and was in high LA pressure shorter in PV scar hearts than control hearts (*n* = 7 vs. *n* = 9, respectively). A refractory period was not reached in 2 PV scar hearts (cf. *n* = 7). Therefore, a mixed-effects linear model was performed. **(B)** In high LA pressure, there was a tendency toward a shorter S1–S2 coupling interval causing AF in hearts with PV scar compared with control (*n* = 6 vs. *n* = 4, respectively, Mann–Whitney *U* test). S1 stimulation (without a premature stimulus) also caused AF. **(C)** The PV activation time (range in the activation time within the PV area including both LPV and RPV) during sinus rhythm was unaltered by the presence of a PV scar as tested with a mixed-effects linear model (*n* = 9 vs. *n* = 8, PV scar vs. control. Electrical noise disturbed the electrograms in 1 control heart, and this recording was omitted from the analysis).

The refractory period was measured in stimulations without AF. In high LA pressure, the refractory period was shorter in PV scar than in control hearts (178 ± 39 vs. 235 ± 48 ms, *p* = 0.011; Figure 7A). In high LA pressure, we observed a tendency toward shorter premature stimulation S1–S2 coupling interval causing AF in hearts with PV scar compared with control hearts [123(66) vs. 174(21) ms, respectively, *p* = 0.067; Figure 7B].

### Site of Stimulation and PV Activation Time

The site of stimulation (RPV or LPV) leading to AF did not change with the presence of localized PV scar (RPV in 1 vs. 2, LPV in 5 vs. 1, both RPV and LPV in 1 vs. 1 PV scar and control hearts, respectively, *p* = 0.325).

The PV activation time during sinus rhythm was similar in PV scar and control hearts (low LA pressure: 32 ± 8 vs. 36 ± 16 ms, and high LA pressure: 32 ± 11 vs. 36 ± 7 ms, respectively, PV scar effect *p* = 0.383, LA pressure effect *p* = 0.900, Figure 7C).

## Discussion

We observed that localized scar in the RPV of otherwise healthy sheep increased the inducibility of AF in high LA pressure. This was associated with a reduction in tissue excitability and shortening in refractoriness compared with control hearts. Moreover, the larger heterogeneity in PV excitability and the stimulation in either RPV or LPV being equally arrhythmogenic suggest electrical remodeling remote from the PV scar.

### Increased Atrial Pressure and AF Inducibility

A large atrium is an independent risk factor for AF (Vaziri et al., 1994), and AF patients often have atrial and PV enlargement (Henry et al., 1976; Tsao et al., 2001). Acute stretching of the atrial myocardium by volume overload increases the inducibility of AF in both AF and non-AF patients as well as in multiple healthy animal models (Thanigaimani et al., 2017). We observed that a high LA pressure increased AF inducibility by stimulation in the PVs in both sheep hearts with and without a PV scar (LA pressure effect: *p* = 0.002; Figure 4B). Others have shown that the stretching of isolated rabbit PVs accelerates spontaneous activations (Chang et al., 2007). The activation of potassium-selective stretch-activated channels causes the hyperpolarization of the resting membrane as well as the shortening of the action potential of myocytes dependent on the timing of the stretch stimulus (Peyronnet et al., 2016). This favors reentrant activation (Moe et al., 1964). In fact, blockade of the stretch-activated ion channels by tarantula peptide shortens the duration of AF induced by burst pacing (Bode et al., 2001).

### Heterogeneous Stretch and Arrhythmogenesis

Increased atrial pressure causes heterogeneous shortening in atrial refractoriness (Ravelli and Allessie, 1997) as well as heterogeneous prolongation in atrial conduction (Eijsbouts et al., 2003). Satoh et al. have demonstrated that an increase in atrial pressure leads to the heterogeneous stretch of the atrial wall (Satoh and Zipes, 1996). AF scroll waves are found to form and linger in interfaces between thin and thick atrial myocardium with heterogeneous stretch in an *in silico* study (Yamazaki et al., 2012). Therefore, heterogeneity in the stretch by LA pressure increase causes a proarrhythmic electrical heterogeneity in the atria.

We induced a localized PV scar and thereby likely induced a heterogeneous stretch because a mature scar is more rigid than the myocardium (Gupta et al., 1994). Ashikaga et al. (2005) observed abnormal mechanics by loss of the transmural gradient in strain beyond the area of ventricular infarction scar in dogs. The resulting mechanical heterogeneity may be proarrhythmic. In fact, a lesser longitudinal strain in the vital border zone of ventricular infarction scar is associated with higher inducibility of ventricular tachycardia in patients (Bertini et al., 2010). Similar mechanisms may occur in the atrial-PV junction with the presence of a PV scar.

We surmised that, in the presence of PV scar, an increase in LA pressure would lead to larger heterogeneity in the wall deformation, to more heterogeneity in excitability, to electrophysiological changes remote from the scar site, and, therefore, to increased inducibility of AF.

### Pulmonary Vein Arrhythmogenesis and Ablation

Initiation of paroxysmal AF depends on premature activation in the PV myocardium in patients (Haissaguerre et al., 1998), whereas atrial remodeling is likely needed for the long-term maintenance of AF (Wijffels et al., 1995). We showed that a localized PV scar provides a substrate for the short-term AF that is likely of reentrant origin. In the presence of tachycardia-induced atrial remodeling, this may lead to chronic AF. Atrial remodeling was absent in our model, and no gross structural abnormalities were observed on CMR. Also, a spontaneous occurrence of PV ectopy was not recorded. Thus, our data suggest that reentrant (intramural) activation in the atrial–PV junction underlies the first cycles of the arrhythmia, especially in the presence of elevated LA pressure. The reentrant activation is likely maintained in the body of the atria as extensive structural and functional remodeling develops in the atrial myocardium.

Our data imply that patients with a PV scar are more at risk to develop AF if they also have increased LA pressure. In fact, a large LA, high LA pressure, and comorbidity of hypertension are known risk factors for PV ablation failure in AF patients (Themistoclakis et al., 2008; Evranos et al., 2016; Sramko et al., 2017). In view of our observations, recurrence of AF after PV ablation may not be interpreted as the disappearance of an antiarrhythmic effect but rather as the appearance of a proarrhythmic PV scar. We speculate that the reduced ablation success rate in these patients can be improved by concomitant pharmacological treatment of comorbidities causing atrial stretch or by extensive ablation to mechanically homogenize the atrium and the PVs.

## Conclusion

A localized PV scar increased the inducibility of AF during increased LA pressure. This was associated with remote electrical remodeling. The proarrhythmic mechanical changes induced by local ablation scar during increased atrial pressure may be the reason for the lower ablation success rate in AF patients with high LA pressure.

## Data Availability Statement

The raw data supporting the conclusions of this article will be made available by the authors, without undue reservation.

## Ethics Statement

The animal study was reviewed and approved by the local ethical authorities at University of Bordeaux, France (approval number 7995) and was carried out in accordance with the EU Directive 2010/63/EU for protection of animals used for scientific purposes.

## Author Contributions

LG, FV, EA, CB, VL, MC, DE, JN, BB, LD, and RC performed the material preparation, data collection, and analysis. LG wrote the first draft of the manuscript. All authors commented on the previous versions of the manuscript. All authors contributed to the conception and design of the study and read and approved the final manuscript.

## Conflict of Interest

The authors declare that the research was conducted in the absence of any commercial or financial relationships that could be construed as a potential conflict of interest.

## Publisher’s Note

All claims expressed in this article are solely those of the authors and do not necessarily represent those of their affiliated organizations, or those of the publisher, the editors and the reviewers. Any product that may be evaluated in this article, or claim that may be made by its manufacturer, is not guaranteed or endorsed by the publisher.
